# Civic Ecology Uplifts Low-Income Communities, Improves Ecosystem Services and Well-Being, and Strengthens Social Cohesion

**DOI:** 10.3390/su13031300

**Published:** 2021-01-27

**Authors:** Rashieda Davids, Mathieu Rouget, Margaret Burger, Kirsten Mahood, Ntswaki Ditlhale, Rob Slotow

**Affiliations:** 1 School of Agricultural, Earth and Environmental Sciences, University of KwaZulu-Natal, Pietermaritzburg, KwaZulu-Natal 3201, South Africa; 2 EnviroHeart Consulting, Environmental and Sustainability Division, Johannesburg, Gauteng 2198, South Africa; 3 Centre for International Cooperation in Development-Oriented Agronomical Research (CIRAD), Plant Populations and Bioaggressors in Tropical Ecosystems Joint Research Unit (UMR PVBMT), 97410 Saint-Pierre, France; 4 Ecolmvelo, Durban 4001, South Africa; 5 Triple-P NPC (Previously i4WATER), Durban 4001, South Africa; 6 School of Life Sciences, University of KwaZulu-Natal, Pietermaritzburg, KwaZulu-Natal, Scottsville 3209, South Africa; 7 Department of Genetics, Evolution and Environment, University College London, London WC1E 6BT, UK

**Keywords:** ecosystem services, environmental management, stewardship, social ecology, social–ecological system, sustainable development

## Abstract

Ecosystem services enhance well-being and the livelihoods of disadvantaged communities. Civic ecology can enhance social–ecological systems; however, their contributions to ecosystem services are rarely measured. We analysed the outcomes of civic ecology interventions undertaken in Durban, South Africa, as part of the Wise Wayz Water Care programme (the case study). Using mixed methods (household and beneficiary (community members implementing interventions) surveys, interviews, field observations, and workshops), we identified ecosystem service use and values, as well as the benefits of six interventions (solid waste management and removal from aquatic and terrestrial areas, recycling, invasive alien plant control, river water quality monitoring, vegetable production, and community engagement). Ecosystem services were widely used for agriculture, subsistence, and cultural uses. River water was used for crop irrigation, livestock, and recreation. Respondents noted numerous improvements to natural habitats: decrease in invasive alien plants, less pollution, improved condition of wetlands, and increased production of diverse vegetables. Improved habitats were linked to enhanced ecosystem services: clean water, agricultural production, harvesting of wood, and increased cultural and spiritual activities. Key social benefits were increased social cohesion, education, and new business opportunities. We highlight that local communities can leverage natural capital for well-being and encourage policy support of civic ecology initiatives.

## 1 Introduction

The magnitude of human activities has pushed us into the epoch of the Anthropocene, where we risk crossing planetary boundaries that would cause catastrophic and irreversible environmental changes, with negative consequences for human well-being [1]. It is predicted that anthropogenic environmental pressures will intensify in the future, resulting in further environmental degradation, climate change, and pollution, and impacting on the ability of natural capital to provide ecosystem services [1–3]. Ecosystems and their services, or “nature’s contributions to people (NCP)” [4], are essential to support human well-being and development [2]. It is understood that natural capital underpins social, human, and built capita, and the interaction between these various forms of capital will determine the levels of well-being that humans could achieve in a particular context through, for example, ecosystem services [5]. Ecosystems and people are interdependent and intertwined through the concept of social–ecological systems.

Social–ecological systems research looks at the reciprocal interactions between people and nature at various temporal and spatial scales [6]. Knowledge of social, ecological, and other components in a system, and on the use and benefit of ecosystem services, is needed in order to derive maximum benefit from interactions in a system. Social–ecological systems provide a basis for understanding the interlinked dynamics of environmental and societal change [6]. Since human activities are the major drivers in social–ecological systems, whereby they can either diminish or enhance ecosystem services and well-being [7], societal change would be essential to ensure ecosystem service protection and sustainability [8]. To foster societal change towards support for environmental management, we need an understanding of how biodiversity and ecosystem services are perceived by humans. Such perceptions would include the way in which humans observe, value, understand, and interpret biodiversity and ecosystem services [9].

Demands for ecosystem services are increased with increasing populations in cities [10], particularly in cities of the global south, that have added pressures of poverty, and direct dependence on ecosystem services for livelihoods and well-being of the poor [11,12]. Ecosystem services provide the foundation for economic opportunities to empower the disadvantaged [2]. The disruption of social–ecological linkages can have detrimental effects on communities, particularly when access to ecosystem services are denied [13], or when ecosystem disservices, such as floods or invasive species, are experienced. This raises the importance of understanding and strengthening social–ecological linkages, while ensuring that ecosystem services are managed appropriately, particularly in disadvantaged communities.

Civic ecology initiatives, or “community-based conservation”, aim to provide diverse environmental and socio-economic benefits through people-centred participatory approaches [14]. Civic ecology practices include environmental stewardship actions that enhance natural capital, ecosystem services, and human well-being, in social–ecological landscapes, such as cities [7]. While civic ecology practices are increasing and contributing to global sustainability initiatives, their contributions to ecosystem services are rarely measured [7].

**Figure 1:**
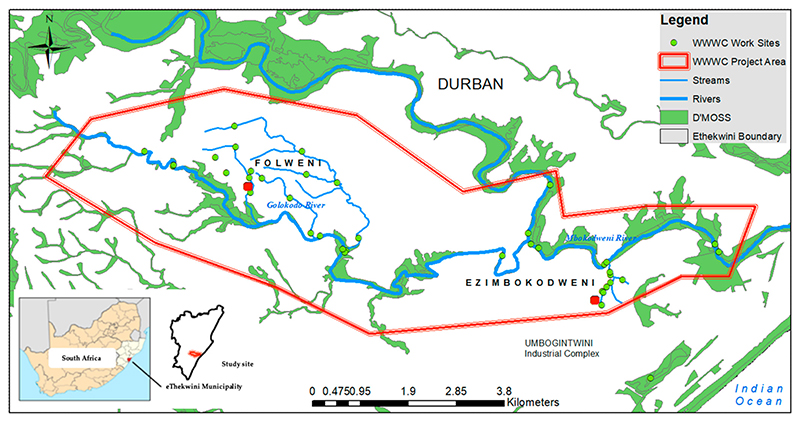
Study area: Wise Wayz Water Care Work (WWWC) sites in eThekwini Municipality (Durban), South Africa, indicating the sites within the peri-urban/rural Ezimbodweni and more urban Folweni communities. D’MOSS—Durban Metropolitan Open Space System.

In this study, we examined the understanding, use, and values of ecosystems and their services with regards to two low-income local communities, one peri-urban/rural and one urban, where some community members are implementing civic ecology initiatives. As a case study, we used the private sector-funded Wise Wayz Water Care (WWWC) programme, being implemented along the Golokodo and Mbokodweni Rivers, within Durban, South Africa (Figure 1). Using a mixed methods approach (household surveys, interviews, field observations, workshops), we investigated the following questions: (1) What are the values and perceptions held by the beneficiaries (people from the community working as part of the WWWC civic ecology programme), and the broader community, related to the WWWC civic ecology programme? (2) What are the various benefits of civic ecology practices to the social–ecological system of disadvantaged communities, particularly with respect to ecosystem services? (3) How do ecosystem services uses and values differ between the beneficiaries and the broader community? In answering these questions, we explored how increased knowledge of ecosystems through civic ecology practices in social–ecological systems contribute to the protection and increased use and benefit of ecosystem services, both for beneficiaries and other members of disadvantaged communities.

## 2 Materials and Methods

### 2.1 Study Area

#### 2.1.1 Socio-Economic Characteristic

The WWWC work area, the study area (Figure 1), is situated in two peri-urban communities, Folweni and Ezimbokodweni, located in Durban, in the province of KwaZulu-Natal, South Africa. Both fall within the eThekwini Metro Municipal boundary. Folweni is more urban and is administered by eThekwini Municipality, while Ezimbokodweni is more peri-urban/rural and is jointly administered by eThekwini Municipality and Ingonyama Trust Board (traditional authority of communally owned rural lands). The study area is characterised as one of the poorest in Durban, with low education, employment, and income levels. In Folweni, 17% have no source of income and 37% earn less than ZAR 1600 (USD 99.60 @ USD 1/ZAR 16.06) per month, 35% have secondary education, only 6% have higher education, 53% of households have piped water inside the dwelling, 42% have flush toilets connected to a sewer, and 47% of households are headed by females [15]. Similarly, in Ezimbokodweni, 20% have no source of income, a third of the population earn less than ZAR 1600 per month, 30% have completed secondary education, only 2.8% have higher education, 10.7% households have piped water inside the dwelling, 4% have a flush toilet connected to a sewer, and 40% of households are headed by females [15].

Sewage infrastructure in the Folweni area is poorly maintained; most of Ezombokodweni utilises informal pit latrines, and is not serviced by waterborne sewer systems, with sewerage being noticed to surcharge into water courses in both areas [16]. A small number of households in Ezimbokodweni are located within the 1:100 floodplain of the Mbokodweni River. Solid waste is a problem, and smaller streams have become blocked by solid waste, invasive alien plants, and illegal sand mining, resulting in stagnant water that exposes the community to various water borne diseases [17]. Issues in the broader area, as noted in the Local Area Plan, include sanitation being a major problem (with failing and unhygienic ventilated improved pit latrines), lack of recreational facilities and meeting venues, lack of tertiary educational facilities, and poor/lack of housing facilities [18].

#### 2.1.2 Bio-Physical Characteristics

The climatic condition of the study area is moderate, situated in a coastal climatic zone, with mean annual temperatures of between 18.5 and 22 °C and a mean annual rainfall ranging between 820 and 1423 mm. The study site is traversed by the Mbokodweni and Golokodo rivers, which fall within the U60E quaternary catchment and the North Eastern Coastal Belt aquatic ecoregion [19]. Numerous wetlands and drainage lines are present along the rivers (Figure 1). River flows, widths, and depths vary across the study area, and between wet and dry seasons. Sites along the Golokodo River are up to 10m wide and 1m deep, and flows range from slow, to moderate, to fast. River substrates include sand and bedrock. Along the Mbokodweni River, widths and depths range from 3 to 20 m and 0.5 to 2 m, respectively, with moderate to fast flows. The dominant substrate is sand, bedrock, and cobble [17].

Results from biological monitoring of Durban’s aquatic systems revealed that 71 of the 175 sites are considered to be in a poor state, and only 3 sites are in a near natural state [20]. Impacts on rivers include illegal spills and discharges, solid waste dumping, sand mining, poor operation of wastewater treatment works, realignment of watercourses, flow reduction, removal of riparian flora, and infestation by invasive alien plants [20]. The rivers in the study area are similarly classified as being impacted by solid waste pollution, bank and channel modification, and invasive alien plant invasion [17,21].

All of the sites are found in the KwaZulu-Natal Coastal Belt vegetation type, within the Indian Ocean Coastal Belt Bioregion [22]. This vegetation type is classed as endangered. Vegetation of significance is situated on settled areas, and along riverbanks, characterised by small valley forests and bushes. In the broader study area, vegetation included small patches of grasslands, many of which have been degraded due to settlement and subsistence farming activities [23].

The site is traversed by the Durban Metropolitan Open Space System (D’MOSS), and parts of the site are classified as Critical Biodiversity Areas [23]. D’MOSS is a formal municipal planning policy instrument that identifies a series of interconnected open spaces that incorporate areas of high biodiversity value and natural areas [20], with the purpose of protecting the globally significant biodiversity (located within the Maputo-Pondoland Biodiversity Hotspot) and ecosystem services within the city [24,25].

### 2.2 Case Study: Wise Ways Water Care Programme

The Wise Wayz Water Care (WWWC) programme commenced in 2016 and brought together community members from Folweni and Ezimbokodweni (the “beneficiaries”), who were previously working as separate volunteer groups, mainly performing litter removal along the Mbokodweni and Golokodo river systems. Under WWWC, the beneficiaries are working and learning together, working towards improving the socio-economic and environmental conditions of their communities through the implementation of various environmental management interventions. This work was stimulated by flooding that damaged houses in the lower lying areas during a heavy rainfall event that occurred in 2016. The flooding was exacerbated by solid waste and alien vegetation blockages in the river systems, which resulted in flow and channel blockages that caused localised flooding. The beneficiaries (*N* = 130) include males (*N* = 41) and females (*N* = 87), with various levels of education, ranging from Grade 1 (lowest level of primary education) to Grade 12 (highest level of secondary education), with 1 person having tertiary education.

The WWWC programme is managed by a non-profit organisation, i4WATER, through funding provided by a business operating in the Mbokodweni Catchment, and located in the Umbogintwini Industrial Complex (Figure 1), the African Explosives and Chemical Industry (AECI) Community Education and Development Trust, since 2016. The objectives of the WWWC programme include improving the environmental health of the lower Mbokodweni Catchment (the study area) and supporting sustainable livelihoods of beneficiaries as well as the greater community through training and skills development, alongside small enterprise development. Beneficiary training included invasive alien plant (IAP) identification, removal, and control; poultry and vegetable production (fertilisation, disease, and pest control; irrigation, harvesting, and marketing); environmental and aquatic management and monitoring (e.g., use of water-related citizen science tools, i.e., miniSASS,clarity tube, *Escherichia coli* (*E. coli*) swab); health and safety training; and community education and engagement.

The beneficiaries of the WWWC programme implemented six environmental management interventions within natural areas in and around Ezombokodweni and Folweni, namely, (1) Solid waste management and removal: removal of waste from aquatic and terrestrial areas; (2) Recycling: waste collection and storage for recycling; (3) Invasive alien plant control: identification and control of invasive alien plants along rivers and streams; (4) Water quality monitoring: monthly biophysical monitoring of river water quality; (5) Community vegetable gardens: vegetable production (two gardens) using permaculture methods; (6) Community engagement: door-to-door community engagement, surveys, and knowledge sharing. Interventions were identified by beneficiaries in response to related challenges faced in the community, and were implemented with support from business funding, within the lower Mbokodweni catchment, at 20 sites, within Folweni (11) and Ezomkodweni (9), along various rivers, tributaries, wetlands, and open areas (Figure 1).

Interventions considered in this study were undertaken over a 3-year period from 2016 to 2018. The removal of solid waste from the rivers took place 4 days per week by 45 team members who managed to collect an average of 1.1 tons of solid waste per month. The recycling team collected and separated the recyclable waste from the collected solid waste, which amounted to approximately 0.48 tons of recyclable waste per month. The community engagement and education team, of 44 members, visited homes in their areas 3 times per week to discuss the various socio-economic and environmental issues that the community is facing. The team also provided information and education to the homes they visited on how to address some of the challenges. The invasive alien plant clearing teams worked along 6.8 km of rivers, as well as in wetlands, to remove invasive alien plants. The team cleared 40 ha using mechanical methods. Species cleared included up to 28 species categorised as invasive in South Africa, primarily *Diplocyclos palmatus*, *Canna indica*, *Arunda donax*, *Lantana camara*, *Melia azerdarach*, *Tithonia diversifolia*, and *Ricinus communis*. The aquatic monitoring team conducted assessments at 22 sites on a monthly basis, analysed and interpreted the data collected, and used the findings to address the challenges undermining the river health. In the 2 community vegetable gardens, 28 team members worked daily to plant a variety of vegetables and herbs, including spinach, tomatoes, carrots, cabbage, kale, beetroot, and lettuce.

### 2.3 Identifying Values and Perceptions of the WWWC Programme

#### 2.3.1 Focus Group Meetings, Workshops, and Interviews

In order to obtain more details on the operational aspects of the interventions, and to ascertain personal perceptions on the programme, we conducted focus group meetings with the WWWC implementers, i4Water, and 1 AECI representative, which involved open discussions of the WWWC programme. We also hosted 2 workshops with 20 and 60 WWWC beneficiaries. During the first workshop, beneficiaries were asked to participate in various individual and group activities in order to (1) identify the positive and negative events or aspects of the WWWC project; (2) identify strengths, weaknesses, opportunities, and threats related to the WWWC programme; and (3) note any changes in the community and biophysical environment that occurred due to the WWWC programme. Personal interviews were held with 9 beneficiaries and 1 coordinator from the programme funding institution in order to obtain greater insight into the WWWC programme, personal experiences, and the manner in which the programme had changed individuals’ lives, including contributions to their livelihoods, sense of place, and health.

#### 2.3.2 Surveys

We conducted surveys (*N* = 3) with beneficiary, community, and external stakeholders (including the WWWC funders, AECI, and government stakeholders (eThekwini Municipality), as well as the South African National Biodiversity Institute (SANBI) (Data S1), in order to identify individual understanding and perceptions of the WWWC programme and associated benefits to the community and beneficiaries, as well as the environment and ES use, and also to gather data on the social, ecological, and economic attributes of the study area [26]. These surveys also collected socio-economic and health data of participants. Open-ended questions were designed to extract perceptions of the value of the programme to the social–ecological-system of the study area. The three surveys were (1) beneficiaries survey, (2) community survey, and (3) key stakeholder online survey. Beneficiary surveys were conducted in a workshop setting (*N* = 60), community surveys were conducted at random households along the Mbokodweni and Golokodo rivers (*N* = 60), and key stakeholder online surveys were conducted via Survey Monkey (*N* = 6). The beneficiary and community questionnaires were translated into IsiZulu, and participants were allowed to choose the language of their preference to complete the questionnaires. Informed consent to utilise the outcomes of the study for research purposes was obtained from all participants, as required by the Ethical Approval. Data collected via the surveys were analysed using Statistical Package for Social Sciences (SPSS) 25. This study is limited in that surveys were only conducted after interventions were implemented.

#### 2.3.3 Site Visits

The authors conducted site visits to Folweni, Ezimbokodweni, and selected WWWC work sites to identify the general living conditions of the community in the study areas (housing, water supply, waste management, etc.), and the biophysical condition of the areas where the WWWC interventions were implemented (wetlands and rivers, open spaces, etc.). Direct field observations were made, and photographs were taken for record purposes. We held on-site discussions with i4WATER and beneficiaries from each of the intervention teams. These visits were done to gain a deeper contextual understanding and gather firsthand data on the interventions and their impacts on site.

#### 2.3.4 Social–Ecological System Workshops with Beneficiaries

In order to better understand the social–ecological system of the study area, we hosted the second workshop with WWWC beneficiaries (*N* = 60), who were randomly selected from the list of beneficiaries. We used A0 size maps as the focus of discussions, which showed the locations of WWWC work areas (WWWC programme boundary and locations of management intervention sites, e.g., water quality monitoring points, and solid waste removal sites). Maps were drawn using ArcGIS 10.4, showing the WWWC work sites relative to other landscape attributes and ecological habitats, namely, the D’MOSS, including wetlands, rivers, and vegetation habitats. Beneficiaries reflected on the maps and related their experiences in the study area. Key questions that were explored in the workshop related to existing or perceived understandings of (1) opportunities related to social activity, knowledge sharing, and natural resource use (e.g., water extraction, livestock grazing, and watering); (2) potential expansion of WWWC work areas; and (3) threats relating to health and safety, such as sources of pollution and illegal dumping of solid waste.

### 2.4 Identifying Ecosystem Services Used and Valued

Ecosystem services were identified from survey responses on the basis of the existing use or demand for that service. Surveys (as described above) were used to collect data on ecosystem service usage by (access), and values of, beneficiaries and community members. The ecosystem services included in the survey were (1) *River water use*: use of natural water from river or stream (e.g., for washing clothes or cars, or for general household use); (2) *Natural material harvesting*: gathering natural materials for various uses, e.g., medicinal plants or wood; (3) *Subsistence use*: direct use of natural resources to sustain life, e.g., food or water; (4) *Agricultural use*: crop or livestock production; (5) *Cultural practices*: use of natural areas for cultural practices or rituals; and (6) *Recreation and leisure:* use of natural areas for leisure or outdoor activities.

## 3 Results

### 3.1 Perceived Ecological, Health, Safety, and Socio-Economic Benefits from Civic Ecology Interventions

**Figure 2:**
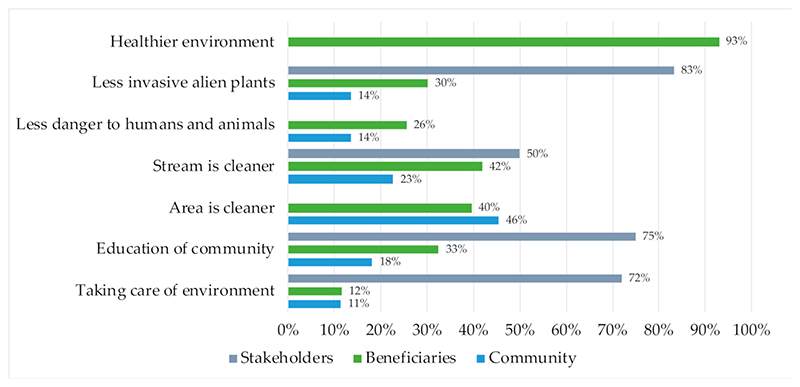
Beneficiary (*N* = 60), community (*N* = 60), and stakeholder (*N* = 6) perceptions of social–ecological changes due to WWWC (stakeholders ranked their agreement with the changes as either minor, moderate, or high achievement, and scores above indicate the weighted average).

Both the beneficiaries (from survey and workshops) and the broader community (from household surveys) reported positive changes in the community after civic ecology interventions had been implemented (Figure 2). These were in the observation that the area and stream were cleaner, but also indirect benefits such as improved education and less danger. Beneficiaries also identified the benefit of improved health, including having noticed a decrease in the number of mosquitos in the area due to the improvement in the river water flow.

The benefit that was most noted by community participants and beneficiaries was that the area was cleaner after clearing solid waste pollution from the land and rivers. This work, coupled with the knowledge sharing on the dangers of littering and poor waste management by beneficiaries, has resulted in a reduction of dumping by residents. This cleanliness can be linked to a decrease in the risk of diseases associated with pollution, and reduction in risk of injury to humans and animals (e.g., reports that skin rashes no longer occurred after children played in the river, and a reduction in mosquitos), which are considered to be positive health outcomes [27].

From all the community respondents who reported to consume vegetables in the survey, more than half of the vegetables consumed were purchased from the WWWC, which shows that the programme provided a significant source of vegetables to the community. This has a positive impact on nutrition through facilitating improved access to a wider variety of fruit and vegetables, resulting in a more balanced diet, with positive effects on health and well-being [28]. WWWC vegetable irrigation was solely from river water.

The community held knowledge of the different programmes being undertaken by the WWWC. Most of the community respondents heard about or interacted with the community engagement (88.2%), invasive alien plant (IAP) control (64.7%), solid waste removal and management (58.8%), vegetable gardening (54.9%), recycling (49%), and river water quality monitoring (23.5%) teams. All respondents who noted the area being cleaner also had knowledge of all the WWWC programmes, showing that community members could relate the work being done by beneficiaries to the positive changes taking place in their community. Comments made in the survey indicated that beneficiaries were appreciated by the community for the knowledge that they shared with respect to environmental education and management.

Half of the external stakeholders, and over 40% of beneficiaries noted that the stream was cleaner after the programme was operational (Figure 2). Over 80% of stakeholders and one-third of beneficiaries noted that there was a decrease in invasive alien plants since the interventions were implemented. This was also visible from site observations (see Figure S1).

**Figure 3:**
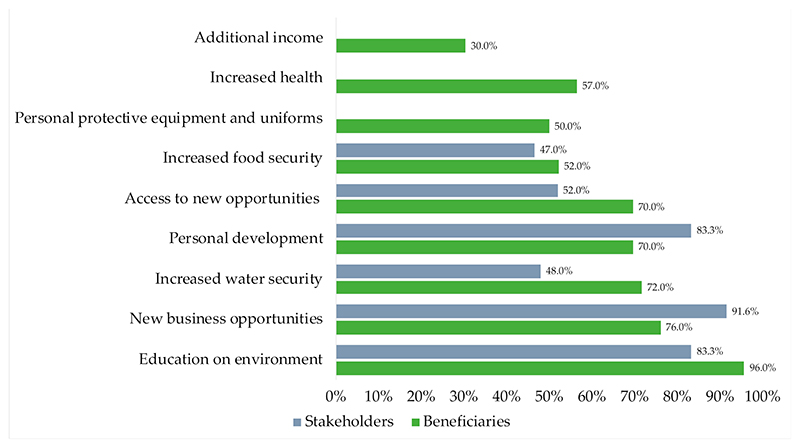
WWWC programme benefits experienced by beneficiaries and perceived by beneficiaries and stakeholders (PPE: personal protective equipment) (stakeholders ranked their agreement with the benefits as either minor, moderate, or high achievement, and scores above indicate the weighted average).

Of the nine benefits beneficiaries experienced from working as part of the WWWC (survey) (Figure 3), more than 60% of beneficiaries experienced six or more benefits, with 96% of beneficiaries listing education on the environment as a benefit, followed by new business opportunities (76%), and increased water security (72%). The first formalised community-based small business was developed by some of the beneficiaries, Envirocare Management Systems (Pty) Ltd., providing prospects for income through invasive alien plant control and water quality monitoring services. External stakeholders similarly perceived the benefits to beneficiaries as high, with 83% noting increased education, 92% noting increased business opportunities, and 83% recognising personal development as benefits to beneficiaries (Figure 3).

From the nine personal interviews that were conducted with WWWC beneficiaries, it was apparent that the WWWC programme had a positive impact on all nine individuals in terms of personal development through education and training, feelings of self-improvement, and increased hope for the future (see Data S2a,b). WWWC also experienced some challenges related to cost recovery, entry requirements for training courses, and illegal dumping (see Data S2c).

An aspect of success that served to encourage sustainable participation in civic ecology initiatives was the increased knowledge, education, and training, which resulted in new skills that benefitted beneficiaries and the broader community, e.g., transitioning from subsistence farmer to small scale producer and undergoing first aid training (Data S2a). Such spin-off benefits to the broader community have strengthened social cohesion.

### 3.2 Nature and Ecosystem Services Enhanced by Civic Ecology Interventions

The natural areas that were enhanced by the interventions included terrestrial and aquatic habitats, e.g., wetlands, rivers/streams, riparian vegetation, and open space (natural areas zoned as public open space). The interventions made positive impacts on ecological areas, and were thus considered to have the potential to enhance ecosystem services. The habitats improved by the interventions are linked to the enhancement of numerous ecosystem services, including regulating services or Nature’s Contributions to People (NCP), of water purification, flood mitigation, biological regulation, and/or disease control, as well as maintenance of biological diversity (genepool protection) (previously considered a supporting service [2], but now captured in regulating NCP [4]); cultural or non-material NCP of aesthetic, recreational, cultural, and education service; and provisioning services or material NCP of water supply, food, and harvesting products [4,29]. People accessed ecosystem services for water, agricultural production, and harvesting of medicinal plants and wood (see Table S1), and increased use of natural spaces for cultural and spiritual activities, since it had been cleaned by the beneficiaries, for example, using the wetland in Ezimbokodweni for cultural rituals (*Umemelo*—Zulu traditional coming of age ceremony for women) (see Figure S1).

### 3.3 Ecosystem Services Uses and Values

**Figure 4:**
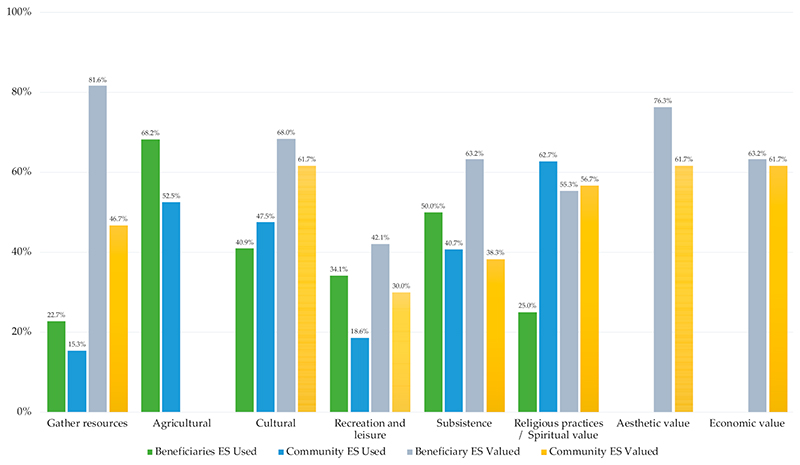
Ecosystem services used and valued by community and beneficiaries. Percentage of respondents that noted they used or valued ecosystem services. *Ecosystem Services Used*: Gather resources—gathering natural materials for use, e.g., medicinal plants, wood; Agricultural use: crop or livestock production; Cultural practices—use of natural areas for cultural practices or rituals; *Ecosystem Services Valued*: Recreation and leisure—use of natural areas for leisure and outdoor activities; Subsistence use—use of natural resources to sustain life, e.g., food, water; Aesthetic value: I enjoy the scenery and beauty of nature; Economic value—I benefit from nature through the sale of products, e.g., traditional medicine, vegetables, wood; Recreational value—I use natural spaces for leisure and outdoor activities. Life sustaining value—it produces goods, and renews air, water, and soil; Spiritual value—natural spaces are valued as being sacred for my religious practices. Cultural value—Natural spaces are important for my cultural practices and rituals and as a place for transferring cultural knowledge through generations; Subsistence value—it provides me with goods to sustain my life, e.g., food and water.

Ecosystem services were widely used and valued by the broader community (randomly selected residents) and beneficiaries (Figure 4). Ecosystem services used most were agricultural use (crop and livestock production), followed by subsistence use (use of natural resources to sustain life), and cultural uses. Beneficiaries valued subsistence ecosystem services the most, followed by aesthetic value and cultural value, while broader community members valued aesthetic, economic, and cultural services the most (Figure 4).

River water was used most for the irrigation of subsistence crops, followed by livestock and personal use (see Figure S2). Participants also used river water for recreation, which was reported to have increased due to the improvement in the cleanliness of the area and the water, since WWWC had been operating. People reported to use the “now clean” river water for washing clothes and cars, as well as for flushing toilets. Business use (by beneficiaries and community members) of river water was for car washing, brick making, livestock, and sales from crop production. More beneficiaries used river water than broader community members for each category. During the workshop, locations of access to ES were reported, including wood and medicinal plant harvesting collection points in adjacent forests, recreational areas, and religious gathering sites. Threats and opportunities related to WWWC operation were also identified (see Table S1). In terms of frequency of river water use by community members and beneficiaries, respectively 28.5% and 40.7% used river water daily, 35.7% and 0% weekly (no beneficiaries reported to use river water weekly), 21.4% and 3.7% used river water monthly, and 14.2% and 48.1% used river water seasonally.

## 4 Discussion

### 4.1 Civic Ecology Contributes to Social–Ecological System Benefits and Ecosystem Service Protection and Enhancement

High use of ecosystem services highlights the importance of natural capital for the livelihoods of people in the community. Similar to other studies, ecosystem services were widely used and valued by the community, and even more so by the beneficiaries as a means to enhance well-being through the mitigation of poverty and diversifying household livelihoods, enhance food security and access to nutritious food, enhance health, improve personal safety and security, access clean water and air, and promote social cohesion [2,30,31]. As found in similar studies, civic ecology practices were also initiated in response to a natural disaster (flood in 2016) [32]. In so doing, the beneficiaries were able to mitigate ecosystem disservices, through environmental management and enhancement of ecosystem services. This led to positive outcomes for both the beneficiaries and their communities [33].

This study confirms that civic ecology practices contribute to the provision of a variety of ecosystem services, including cultural services such as education and learning, social relations, and recreation [7]. We confirmed links between spiritual values and resource management [34], whereby management, environmental protection, and stewardship, increase when people associate spiritual and cultural value with natural areas [35].

The social–ecological interactions in the community influence the manner in which people value the environment, whereby valuation of biodiversity is determined by the practical function obtained from the ecosystems and ecosystem services that enhance the livelihoods of individuals [36]. The perceptions of values identified in this study assert that there is strong dependence of people on ecosystem services, and their understanding of this dependence has, in turn, motivated them towards voluntary environmental stewardship.

We confirm that civic ecology practices both sustain human health [37] and lead to the creation of new natural capital [38]. Our study supports the understanding that local communities can benefit from projects that aim to integrate sustainable development and environmental management, and can create positive attitudes and perceptions towards conservation initiatives [39]. Such projects should aim to incorporate the environmental, social, and economic dimensions, including sustainable use of ecosystem goods and services, promoting dignified standards of life, and providing employment opportunities [39].

The results have governance implications. The interventions were able to address some of the impacts on Durban’s rivers [20] and enhance terrestrial habitats within Critical Biodiversity Areas that are crucial to meet biodiversity targets [40], thereby reducing the pressure on government authorities who are mandated to manage these areas for conservation purposes. The outcomes of this study related to ecosystem service uses by disadvantaged communities can also be considered by authorities in preparing conservation plans, where such understanding may assist in determining the capacity of ecosystems to support both social and ecological communities [26]. This study highlights that local communities can leverage natural capital for well-being and social-ecological improvements and encourages policy support of civic ecology initiatives.

### 4.2 Civic Ecology Provides Opportunities for Social Cohesion and Personal Development

We show that social cohesion is critical for the achievement of sustainability and well-being [2], and that ecosystem services provide a basis for spiritual, cultural, and social cohesion experiences [4]. Such perceptions, when coupled with scientific evidence of positive outcomes of management interventions, provide a powerful combination for ensuring the sustainability of civic ecology programmes.

Positive perceptions of community members of the impacts of environmental management can ensure both support for, and long-term sustainability of, management initiatives [41]. The perceptions of the direct relationships between the positive social–ecological changes taking place in the area and the work being done by the beneficiaries has strengthened social cohesion in the community.

The involvement of the community in the selection and implementation of the interventions strengthened the sustainability of the interventions. Our study provides evidence that, contrary to the notion of the tragedy of the commons [42], by taking ownership and control of natural capital, local communities can successfully contribute to improved collective human well-being.

## 5 Conclusions

Our study showed that increased knowledge of ecosystems through civic ecology practices contributed to the protection and increased use and benefit of ecosystem services, both for beneficiaries and other members of disadvantages communities. Civic ecology practices have the potential to uplift impoverished communities through providing opportunities for education, as well as enhanced ecosystem service protection and access, and should, therefore, be encouraged and supported by government and policy. Given that contributions of civic ecology groups are increasingly recognised by governments for their contribution to natural capital, they need to be supported by the government and the private sector through policies aimed at achieving sustainability and well-being [43].

This study provides evidence of the potential for civic ecology initiatives, supported by private practice, to overcome the tragedy of the commons and enhance ecosystem services for low-income communities who are directly dependent on ecosystem services for their livelihoods and well-being. We call for increased governance support of similar civic ecology initiatives as a means to capacitate local communities to take ownership of natural capital and make gains in the plight against poverty and environmental degradation.

## Supplementary Material

Supplementary materials

## Data Availability

The data presented in this study are available on request from the corresponding author.
