# Supplementary material for: Civic Ecology Uplifts Low-Income Communities, Improves Ecosystem Services and Well-Being, and Strengthens Social Cohesion
Source: Sustainability. Author manuscript; Available in PMC 2023 Sep 8. (PMC7615067; doi:10.3390/su13031300)
Supplement: Supplementary materials [file EMS187383-supplement-Supplementary_materials.pdf]

CIVIC ECOLOGY UPLIFTS LOW-INCOME COMMUNITIES, IMPROVES ECOSYSTEM SERVICES AND WELL-BEING AND STRENGTHENS SOCIAL COHESION

SUPPLEMENTARY MATERIAL

Figures

Figure S1: Ezombokodweni Wetland 2015 (before WWWC) and 2018 (after WWWC)

Figure S2: Natural water used by beneficiaries and community members

Tables

Table S1: Social-ecological system workshop findings

Data

Data S1: Questionnaires/Surveys

Data S2: Stories of change, personal comments, and challenges:

- Data S2a: Stories of change
- Data S2b: Comments made by beneficiaires, community members and external stakeholders.
- Data S2c: WWWC Challenges

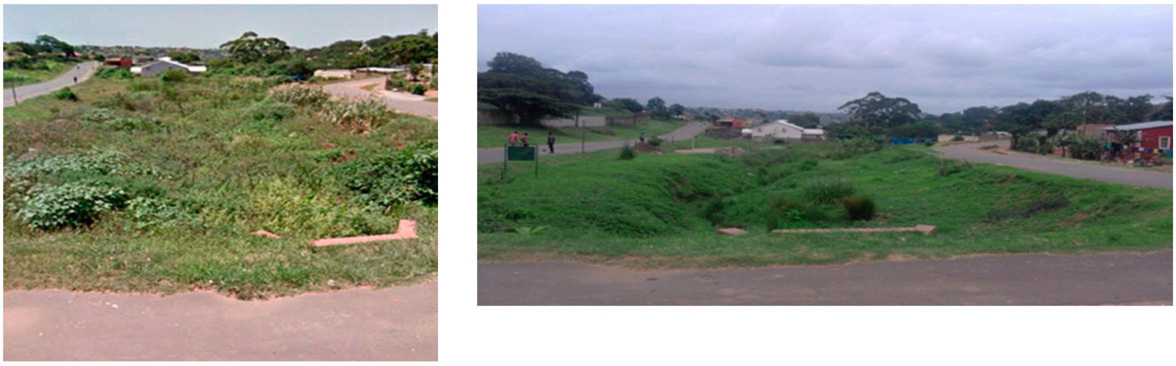

SM Figure S1: Ezombokodweni Wetland 2015 (before WWWC) and 2018 (after WWWC)

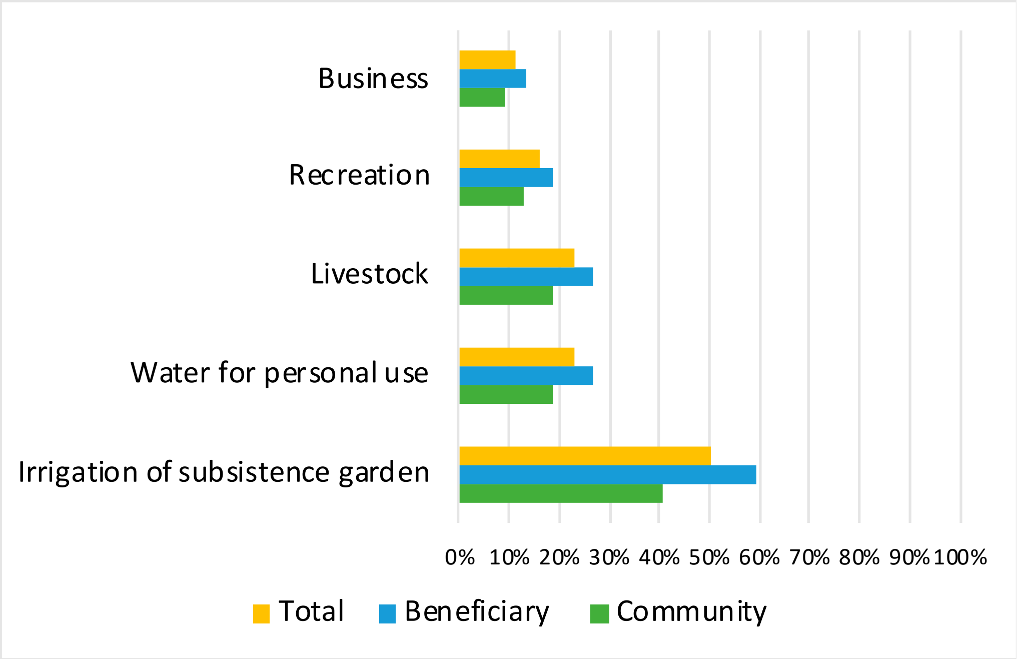

SM Figure S2: Number (%) of repondents who reported to use natural water by category of use

SM TABLE 1

SM Table S1: Spatial analyses workshop: points of interest for ecosystem services, opportunities and threats. During a workshop, beneficiaries identified various points of interest in the landscape where ES were accessed and also noted opportunities and threats related to implementation of interventions

	Folweni	Ezimbokodweni
Ecosystem services	Beneficiaries identified collection points for wood, indigenous plants and herbs in the adjacent D'MOSS thicket and forest areas.<br>The 'Adopt-a spot' where beneficiaries have been working was identified as a space of recreation. It was noted that the community has now started using this spot for cultural activities, including Umemelo ceremony.<br>Locations of where community members were using river water for swimming (children), collection of drinking water for cattle and washing clothes were identified.<br>Locations along the river where animals (cattle) drink water were identified.	A Shembe Temple adjacent to the community hall was noted as an important natural space for religious and cultural activity.<br>Two locations of where community members were collecting water and one where the municipality was collecting were identified.<br>Wetland rehabilitation has taken place which has balanced the ecosystem and also facilitated vegetable gardening through the provision of cleaner water.
Threats	Numerous dumping sites, including scrap metal, rocks and dead animals were identified along the river.<br>Two locations of where illegal sand mining was taking place were identified. Beneficiaries noted that the banks of the stream keep widening in these areas and that the work they are doing there is resultantly compromised.<br>Area under the bridge where the D'MOSS wetland is located, was identified as a blockage point for sand after illegal sand mining activities upstream of the site have occurred.	Two locations of leaking sewer pipes were identified.<br>Pollution from a local business (pot making) including air, noise and water pollution was identified.<br>A site of high infestation of invasive alien plants was identified and linked to crime and illegal dumping.<br>A donga posing danger and safety risk was noted.<br>A stream and wetland where there is sinking mud was identified.

	The area close to a brick making and car wash business was identified as a difficult area to get through (not easy to work there).<br>The WWWC work site next to a tuck shop was noted to have little to no improvement due to recurrent dumping, littering and leaking sewer pipes.	
Opportunities	A Learning Centre, soup kitchen and a Red Cross Community hall were identified as a potential spaces for social networking.<br>'Baba Majola' has been operating a vegetable garden for many years and was identified as a source of indigenous knowledge for growing food.<br>Locations of where other community groups are growing vegetables, working on cleaning the stream and recycling (school) were identified.	The Hukukushu and Thola streams and wetland were identified as areas of opportunity for WWWC to work in.<br>A location of a traditional healer who showed interest in working on the WWWC programme was noted. An alternative location of a traditional healer was also noted to both release 'good and bad spirits' into the community.<br>Two sports grounds and a community hall were identified as areas for social activities.

Data S1: QUESTIONNAIRES

Questionnaire No		Date		GPS Coordinates
WWWC Project/s				
Folweni team (tick)		Ezimbokodweni team (tick)		

1. PERSONAL INFORMATION & HOUSEHOLD DEMOGRAPHICS						
**Name (optional):**			**Address (optional):**			
**Your age:**<br><br>**Your gender (circle): Male**<br><br>**Female**	**Total no. of males in household:**<br><br>**Ages of males in household:**			**Total no. of females in household:**<br><br>**Ages of females in household:**		
**How many people in your household are employed in these categories?**	*Full time:*	*Part-time:*	*Contract:*	*Unemployed:*		*Pensioners:*
**What type of dwelling you live in?**	Formal brick	Informal	Traditional	Other, please specify:		
**Type of activities are occurring at your household?**	Shop	Crop farming	Livestock farming	Recycling for income	Business (specify)	Other, please specify

**What type of energy sources do you use in your household?**	Electricity	Gas	Wood	Paraffin	Candles	Other, please specify:

2. WATER, SANITATION AND WASTE

**What is the source of potable water for your household?**	Inside pipe	Communal tap	Communal tank	River/stream		Other, please specify		
**What type of toilet do you use?**	VIP toilet	Flushing toilet	Septic tank system	Other, please specify:				
**How do you store your waste?**	Refuse bags	Bin	Communal dump	Communal waste skip		Other, please specify		
**How do you dispose of household waste?**	Collected by municipality (DSW)	Collected by contractor	Take it to waste dump	Recycle	Burn it	River	Other:	
**Which of the following do you practice in your household? (Circle often, seldom or never)**	Re-use of water<br><br>Often<br>Seldom<br>Never	Composting<br><br>Often<br>Seldom<br>Never	Recycling<br><br>Often<br>Seldom<br>Never	Energy conservation<br><br>Often<br>Seldom<br>Never		Other, please specify:		

1. NATURAL CAPITAL & ECOSYSTEM SERVICES

DEFINITIONS OF TERMS

Nature: Living things of the physical world collectively, including plants, animals, the landscape, and other features and products of the earth, as opposed to humans or human creations.
Biodiversity: the variety of different types of plants/trees and animals. (field assistant to explain to respondent)
Environment: the surroundings or conditions in which humans, animals, or plants exist, including physical, chemical and other natural forces.
Ecosystem: an interconnected community of natural living organisms and their physical environment.
Ecosystem services: the goods and services (benefits) we as humans derive from nature / natural systems (e.g. water, air, soil, plants and animals (food).
Conservation: the act of preserving, guarding or protecting an ecosystem or wildlife. (field assistant to explain to respondent)
Invasive alien species: species of plants, animals and other organisms that are non-native to an ecosystem, and which may cause economic or environmental harm or adversely affect human health.

3. NATURAL CAPITAL & ECOSYSTEM SERVICES

	**Nature**	**Biodiversity**	**Environment**	**Ecosystem goods and services**	**Conservation**	**Invasive alien species**	**Frequency**	**Source of information**
**1.Have you heard of the following concept?**	**Yes No**	**Yes No**	**Yes No**	**Yes No**	**Yes No**	**Yes No**	1. Seldom<br>2. Often<br>3. Very often	1. School<br>2. Newspaper<br>3. Television<br>2. Radio<br>5. Wise Wayz Water Care<br>6. Other NGO / community group<br>7. Municipality<br>8. Friends/family<br>9. Pamphlets/brochures
**2.What is your understanding of the term**								
**3.How often have you heard of the following concept? Frequency – see scale.**								

**4. Where did you get this information from? See list of sources of information.**								
**5. If there is an isiZulu or isiXhosa word/s (specify)**								

**Which of the following do you value from the natural environment? (Multiple responses permitted- ask for each)**		
1.**Aesthetic value**: I enjoy the scenery and beauty of nature	2.**Economic value**- I benefit from nature through the sale of products e.g. traditional medicine, vegetables, wood	3.**Recreational value**- I use natural spaces for leisure and outdoor activities
4.**Life sustaining value**- it produces goods, renews air, water and soil for me	5.**Spiritual value**- natural spaces are valued as being sacred for my religious practices	6.**Cultural value**- Natural spaces are important for my cultural practises and rituals and as a place for transferring cultural knowledge through generations
7.**Subsistence value**-it provides me with goods to sustain my life e.g. food, water		

3.NATURAL CAPITAL AND ECOSYSTEM GOODS AND SERVICES

**What do you consider to be part of nature / biodiversity in your community? (Multiple responses permitted- ask for each)**	Gardens *	Open space	Rivers	Insects	Plants/trees	Animals	People	Other
**Please rank the following according to what you consider to be an important part of nature in your community** - Codes: 0=not	Gardens *	Open space	Rivers	Insects	Plants/trees	Animals	People	Other

important 1=slightly important 2=Important 3=Moderately important 4=Very important								
**Which of the following ecosystem services are you aware? (Multiple responses permitted)**	None	Climate control e.g. shade	Flood control	Water supply	Water purification	Sediment retention	Pest control	Other
**What are the main ecosystem services you use?**	Gather resources	Agricultural use	Cultural practices	Recreation and leisure	Subsistence	Religious practices or sites	Other, please specify	
**Are you using river water for personal use, recreation, business or irrigation?**	Personal use. Please specify.	Recreation	Irrigation of subsistence garden	Livestock<br><br>If yes, do your livestock graze in the local area?	4. Business (specify)<br>4a. Car wash<br>4b. Brick making<br>4c. Livestock (for sale)<br>4d. Vegetables /crop (for sale)	Other, please specify:		

*The word Garden in this questionnaire is associated with vegetable gardens in isiZulu as *ingadi yemifino*. Where necessary specify a flower garden as *ingadi yezimbali.*

	Yes / No	Where do you get it and what do you use it for?	How often do you use it? (1 – Daily, 2- weekly, 3- monthly, 4 – seasonally)	Amount (per month in Rands of value to you)
**1.Do you use water that is not from a tap?**				
**2. Do you use natural soil or sand?**				
**3. Do you use animals from your natural area?**				
**4. Do you own animals? If so which animals do you own? (circle)**	Chicken<br><br>Goats<br><br>Cows<br><br>Sheep<br><br>Other	Do your animals use or depend on the natural environment for:<br>Grazing: Yes No<br><br>Water supply: Yes No<br><br>If chickens, what is their source of food?<br><br>If chickens, do you use their manure and what for?		
**5. Do you use insects from your area? If so which insects and what are these used for?<br>4a. Do you eat any insects? Name these.**		If eated, how often?<br><br>Daily<br>Weekly<br>Monthly<br>Yearly		
**7. Do you use plants from your area? If so which plants and what are these used for?**		If eaten, how often?<br><br>Daily		

	**Yes / No**	**Where do you get it and what do you use it for?**	**How often do you use it? (1 – Daily, 2- weekly, 3- monthly, 4 – seasonally)**	**Amount (per month in Rands of value to you)**
**7a. Do you use any invasive alien plants? Please name these.**<br>**7b. Do you use any indigenous plants? Please name these.**<br>**7c. Do you eat any of these?**<br>**7d. Are they used for medicinal purposes?**		Weekly<br>Monthly<br>Yearly		
**Do you use fuelwood from your area?**				
**Do you use clay from your area?**				
**Do you use thatch / logs / wood from your natural area?**				
**Do you use stones from your area?**				
**Other resources you use from nature:**				

**Have you noticed any changes in the quality of the river and when?**	Yes	No	Describe the changes and when they occurred:
**Have you noticed any changes to the natural environment (after the work has been undertaken by the Wise Ways Water Care team?**	Yes	No	Describe the changes and when they occurred:

4 HEALTH AND DIET									
**What are the main food items you consume in your household?**	**Maize**<br><br>Daily<br>Weekly<br>Monthly<br>Never	**Bread**<br><br>Daily<br>Weekly<br>Monthly<br>Never	**Red meat (beef, lamb, etc)**<br><br>Daily<br>Weekly<br>Monthly<br>Never	**Chicken**<br><br>Daily<br>Weekly<br>Monthly<br>Never	**Fish**<br><br>Daily<br>Weekly<br>Monthly<br>Never	**Vegetables**<br><br>Daily<br>Weekly<br>Monthly<br>Never	**Fruit**<br><br>Daily<br>Weekly<br>Monthly<br>Never	**Dairy (milk, cheese, yoghurt etc)**<br><br>Daily<br>Weekly<br>Monthly<br>Never	**Other, please specify:**
**Do you consume vegetables that you grow yourself?**<br><br>**Yes** **No**	**Do you consume chicken that you grow yourself?**<br><br>**Yes** **No**<br><br>**Do you consume eggs from your own chickens?**<br><br>**Yes** **No**		**Do you consume meat or milk from your livestock?**<br><br>**Yes** **No**<br><br>**Goats**<br>**Cows**<br>**Sheep**<br>**Milk**		**Do you exhange/barter and food items with community members?**<br><br>**Yes** **No**<br><br>Daily<br>Weekly<br>Monthly<br>Never		**Do you sell vegetables to the community?**<br><br>Yes No<br><br>Which vegetables:<br><br>**Do you sell livestock to the community?**<br><br>Yes No		

5. WISE WAYZ WATER CARE

**Are you part of any community group?**	Religious	Community safety (neighbourhood watch)	Funeral group	Political	Finacial group (credit / borrowing/stokvel)	Other:
**Are you aware of the Wise Ways Water Care Project?**	Yes	No				
**Which of the Wise Wayz Water Care Projects have you seen, interacted with or heard about?**	Invasive alien plant control and removal	Community engagement on issues of solid waste, water leaks, infrastructure monitoring & education	Community vegetable gardens	Recycling	Water quality monitoring	Solid waste removal from water courses
						None
**What type of benefits do you feel the Wise Wayz Water Care project brings to your community?**	None	Source of income activity (how)	Increased food security	Increased water security	Improved natural environment and rivers	Social upliftment, skills development and education
	Increased water security	Improved health				
**Are there any other benefits you feel the Wise Wayz Water Care project brings to the community?**						

**Do you consume vegetables that you buy from:**	**Do you consume chicken that you buy from:**		**Do you consume meat that you buy from:**		Which items?		Which livestock:
**Community member:** Yes No<br>**WWWC garden:** Yes No	**Community member:** Yes No<br>**WWWC garden:** Yes No		**Community member:** Yes No<br>**WWWC garden:** Yes No				**How often do you slaughter for personal consumption?**<br>**Or for events?**
			**Goats**<br>**Cows**<br>**Sheep**<br>**Milk**				
**Have you or a family member fallen ill after consuming produce from the local community or your own?**	Yes, please note illness:			No			None
**Have you or any family member experienced any ill health after interacting with the natural environment in your area?**	Yes, please explain		No	Don't know			
**If yes to the above question, what was experienced?**	If yes, please specify: Diarrhoea	Skin rashes	Vomiting	Injury	Bilharzia	Worms	Other, please specify:
**Have you noticed a change in the incidence of any of the above health issues? When did the change occur and why do you think the change occurred?**	Yes	No	When	Why			

**What do you feel could be done to avoid the above health issues in your community?**	

**Has the WWWC project affected your views towards the natural environment? How?**	No effect	More caring about the environment	Provided more knowledge about the importance of the environment	Increased sense of pride in community	Other, please specify:

**Do you think the Wise Wayz Water Care project has caused conflict or challenges in the community?**	No	If yes, please specify:
**Do you feel that the WWWC project has shared information on their work in your area adequately?**	No	If yes, please specify:

**Do you have any recommendations improve the Wise Wayz Water Care project?**	None	If yes, please specify:
**Are there any additional comments you would like to share?**		

Data S2a: STORIES OF CHANGE

Summary

One participant had the ambition for tertiary education, but due to a lack of funding, could not pursue his dreams of further education, until he joined the programme. Another felt that her involvement in the programme gave her the ability to make positive contributions to her community, which she would otherwise not have been able to do. A beneficiary also noted that the increase in his knowledge base through education and training has increased his vision and drive to advance his business skills – whereby he is in the process of transitioning from a subsistence farmer to a small-scale producer with aspirations to become a commercial farmer. One beneficiary was proud of the range of course certificates she had obtained through the programme, including plumbing, water safety, safety, health and environment (SHE), invasive alien plant training, and door-to-door training, through which she also gained the opportunity to travel to another city. She felt empowered as a woman doing plumbing work and wanted to "show men that women can do this work!" Similarly, another beneficiary and Director of Envirocare Management Systems (Pty) Ltd, also proudly mentioned the list of training he had received, including invasive alien plant control, SHE, first aid, door-to-door and snake handling. From the interviews, it was clear that being part of the WWWC programme had changed the lives of beneficiaries for the better.

Respondent 1: Male

Respondent 1 is a WWWC Youth beneficiary from KwaMakhutha. He obtained access through the RCU eZimbokodweni group. His learning pathway has been through starting a B Com Marketing, but he was unable to continue due to funding constraints. He has embraced the many opportunities offered within WWWC training and skills development. The training includes Geo ODK, Sinqonqozela Ulwazi (which fits well with his marketing studies), Plant identification, a driver's licence and being part of the Executive. Respondent 1 confirmed that being part of the Executive has enabled professionalism, understanding and application of governance. As part of the WWWC

Executive (acting as secretary) for the last year, he feels he has grown immensely. He was honoured with the Community Builder Award at the 2017-year-end function but remains humble and committed. He feels older people in the programme can teach the younger people and that a balance is required where the generations can learn together. He has focused on environmental learning opportunities. This is substantiated in his Directorship with Envirocare Management Systems (Pty) Ltd, the first formalised small business to grow out of the WWWC programme.

Respondent 2: Female

Respondent 2 matriculated in Umlazi and lives eZimbokodweni with her family. She is part of the RCU group. She has participated in many different types of training such as invasive alien plant control, water safety and Door-2-Door. She is an important member of the Sinqonqozela Ulwazi team and emphasises that she likes this role in assisting community members to understand more about water and the work of WWWC. She added that she loved plants and grows spinach, sugar cane and cabbage at home. The sugar cane is eaten at home for sugar. As she describes her training her knowledge of plants is evident. She mentions Iboza (Tetradenia riparia) and its medicinal use, so too Bugweed and Syringa, isigqikisomkhovu (cycads) and explains she likes indigenous plants the most.

Respondent 3: Female

Respondent 3 is a passionate young woman and hails from eZimbokodweni. She is the Vice Chairperson of RCU and a member of the WWWC Executive. Respondent 3 noted that although she does not earn a salary, she has a vision to improve her long-term future being for herself and her family. She regards WWWC as an opportunity to uplift herself and her children and speaks often of the importance of leaving a legacy. Prior to becoming part of RCU, she trained in security and obtained a welding certificate. She is a member of the African Gospel Church and explains she wants to leave a legacy of her life. It is this legacy that motivates her to participate in all the training that is offered. She has attended a Woman in Leadership training course in Richmond and is currently

undertaking a counselling course as well. She feels that the members of RCU have helped to groom one another and is proud to be part of this group.

Respondent 4: Female

Respondent 4 has been part of Emvelo-wise in Folweni for 11 years. She noted that joining WWWC has changed how she related to growing vegetables. As one of the older more established members of the Folweni Emvelo-wise team, she mentions adding lettuce, (both green and purple) to the spinach, beans, peanuts and cabbage they grow. Cabbage is bought by the local Boxer store at R10.00 each. She mentions the many training opportunities and has participated in First Aid as well as Water Safety.

Respondent 5: Female

Respondent 5 hails from Folweni and is a member of the Emvelo-wise group. She has joined the WWWC Executive where she represents Emvelo-wise. Her biggest change has come through "working as a team, we did not know what to do, we were behind". She has embraced training and has completed poultry production training and now successfully raises 'broilers'. Other training, she mentions is Water Safety, plant identification and invasive alien plant control and herbicide application. The Women's Leadership training she has completed was also proudly mentioned.

Respondent 6: Male

Respondent 6 is from Folweni and a member of the Emvelo-wise group. He felt that WWWC has brought the two teams together and has taught them a lot. He has a desire to share his knowledge. His training has been in vegetable gardening, including the three-month training in Mpumalanga that has changed the way he regards vegetable growing. He mentions aspects such as creating their own

seedlings, land preparation, vegetable storage and sales as well as herbicide and pesticide use. He too is proud of the lettuces they grow as well as the many additional 'new' vegetables not grown before.

Respondent 7: Male

Respondent 7 is from Folweni and a member of the Emvelo-wise team. He stated that "I am going to be a farmer, and Agrobusiness farmer". He explained how attending training in Mpumalanga has increased his vision and desire to advance his business skills. Before he was at 'a ground level and a street vendor', but now he will be able to 'plant more hectares' and become a 'commercial farmer'. He added that WWWC has changed his life.

Respondent 8: Female

Respondent 8 is part of the RCU group and lives in eZimbokodweni. She had completed many of the training courses and noted that "I have many certificates". Certificates in plumbing, water safety, SHE, IAPs and community engagement (Door-to-Door). She valued her association with WWWC as "these opportunities have enabled me to travel to Cape Town and practice the Travel & Tourism I learnt at school". In addition, she explained that her understanding and attitude towards environment has shifted, stating that "WWWC has given me a chance to understand environment". She sees herself in future as a female plumber in a business that "will show the men that women can do this work"!

Respondent 9: Male

Respondent 9 is from Folweni and a member of the Emvelo-wise team. He is also a member of the WWWC Executive. He has completed many different training courses. He mentions, IAPs and PCO, Safety, Health and Environment, First Aid, Community Engagement (Door-to-Door) and snake handling. As part of Emvelo-wise he worked in the streams and the vegetable gardens. He mentioned clean-up campaigns in Folweni and how people should not waste water and litter. He felt

that WWWC has helped to open people's eyes that "impilo or life is connected to the environment and we should not be wasting water at our homes". As a director in Envirocare Management Systems (Pty) Ltd, the first formalised small business to grow out of the WWWC programme, he is a valued member who has a code 10 drivers licence and his own car.

Data S2b: COMMENTS MADE BY PARTICIPANTS ON THE WWWC PROGRAMME

Comments on beneficiary benefits of WWWC made by external stakeholders

"The enthusiasm and subject knowledge displayed by the beneficiaries is a marvel to witness"

"The lives of those young people and their generations will never be the same again."

"I see them growing becoming better people than before, the Project gave them the purpose in life, they are confident, proud on what they are doing, and their generations will never be the same again."

"I have come across comments from beneficiaries whom were doing Adult Education and Training where one elderly lady said it does not matter if I fail, what is important is that I can now write and read my name. To her this was utmost achievement. Another lady made a similar account stating my grandchildren used to rob me of my pension money as I did not know how to count but through AET I can now count, and they are finding it difficult to rob me. I have come across plenty of similar statements."

Comments by beneficiaries on benefits of WWWC

"I now have a reason to get up in the morning, compared to other youth in the community who don't work and waste their time with drugs."

"I just want to thank the way WWWC is moving, it is developing the people."

"WWWC teaches us to be independent and to create more job opportunities with the skills the project provided."

"So far the project has motivated the people to have faith and start their own businesses."

Comments by community members on benefits of WWWC

"I wish for it to grow, because they are our hope now. We would like for our children to join them in future and be exposed to opportunities."

“WWWC had taught me a lot about the importance of the environment, I am thankful.”

“Taking of the environment should be shared with schools too so that the whole Africa can have access to water.”

“Thank you for visiting our home, I have learned a lot and I will pass the knowledge to others”

“We appreciate the work you do.”

Data S2c: PERCEIVED CHALLENGES OF WWWC

Two beneficiaries and six community members (8% in total), noted that WWWC causes conflict in the community. This was related to the perception that some beneficiaries received cost recovery (provided to beneficiaries for carrying out activities in addition to basic activities), while others did not. Another challenge was that some training courses needing a minimum of Grade 9 level secondary school education, which a few of the older beneficiaries did not have. This issue was subsequently addressed by i4WATER through the provision of adult education courses geared towards obtaining the required levels of education for course enrolment. Other challenges that were effecting the progress of the WWWC interventions were continued illegal dumping, especially of used diapers, in certain areas, and sewer pipe leakages into the river (SM Table 1).
